# Eco-exergy and emergy based self-organization of three forest plantations in lower subtropical China

**DOI:** 10.1038/srep15047

**Published:** 2015-10-21

**Authors:** Hongfang Lu, Fangyan Fu, Hao Li, Daniel E. Campbell, Hai Ren

**Affiliations:** 1 Key Laboratory of Vegetation Restoration and Management of Degraded Ecosystems, South China Botanical Garden, Chinese Academy of Sciences, Guangzhou 510650, China; 2 US EPA, Office of Research and Development, National Health and Environmental Effects Research Laboratory, Atlantic Ecology Division, 27 Tarzwell Drive, Narragansett, RI, USA

## Abstract

The bio-thermodynamic structures of a mixed native species plantation, a conifer plantation and an *Acacia mangium* plantation in Southern China were quantified over a period of 15 years based on eco-exergy methods. The efficiencies of structural development and maintenance were quantified through an integrated application of eco-exergy and emergy methods. The results showed that the storage of eco-exergy increased over 3 times in all three plantations, as predicted by the maximum eco-exergy principle. This trend was primarily seen due to the accumulation of biomass, instead of an increase in the specific eco-exergy (eco-exergy per unit biomass), although species richness did increase. The eco-exergy to emergy and eco-exergy to empower ratios of the three plantations generally increased during the study period, but the rate of increase slowed down after 20 years. The dominant trees are the largest contributors to the eco-exergy stored in the plantations, and thus, the introduction of suitable indigenous tree species should be considered after the existing trees pass through their period of most rapid growth or around 20 years after planting. The combined application of C-values and suggested weighting factors in the eco-exergy calculation can imply opposite results, but may also supply useful information for forest management.

Forests cover about 30% of the land surface of the Earth and they are key terrestrial ecosystems for maintaining and adjusting the global ecological balance and for improving environmental quality^1^,^2^,^3^. However, forests are shrinking and degrading rapidly throughout the world due to the continuous increase of human demands for wood products, as well as land for agriculture and urbanization^4^,^5^,^6^. Afforestation is taken as one of the main methods to deal with many environmental problems, such as soil erosion and carbon emissions^7^,^8^,^9^. Clarification of the dynamic mechanisms of forest plantations is a priority for their successful long-term management^10^,^11^. However, past studies have shown complicated, and unsynchronized nonlinear dynamics that cause the development of different trends in the structures and functions of forests^12^,^13^. Consequently, forest management and decision-making has been made even harder, because whole system optimization cannot be obtained by simply maximizing any one of the forest’s structures or functions. A synthetic target factor for forest management is still missing and difficult to agree upon, as evidenced by the attempts to manage the U.S. Forest Service lands in the Pacific Northwest United States for timber production and endangered species habitat preservation, e.g., the spotted owl, from 1994 to 2003^14^.

Energy is the basic driving force for all processes, and all ecological processes are accompanied by the transformations of energy potentials. Therefore, there may exist some general thermodynamic principles behind the complicated structural and functional development of ecosystems^15^,^16^, and exploring them has been a burning issue in both ecology and biophysics, and consequently this interest has resulted in the development of a variety of *orientors*^17^,^18^. Most of these energy orientors are rooted in the maximum power principle (MPP) first proposed by Lotka^19^,^20^, which states that self-organizing systems, especially biological systems, capture and use available energy to develop network designs that maximize the energy fluxes through them that are compatible with the constraints of the environment, and that those systems that maximize power will prevail. Thus, the MPP governs expediencies in both functional and structural development, as examined by Odum^21^.^22^ and Jørgensen & Mejer^23^, respectively.

Based on system and hierarchy theories, H.T. Odum developed the concept of emergy, which was defined as the available energy of one kind previously used up directly and indirectly to make a service or product in all types, i.e. different types of energies, materials and information are expressed on a common basis^24^. Consequently, Odum extended Lotka’s definition of the MPP to consider not only energy flows but also material and information flows of all different types, all objectively converted to emergy flows, and in doing so he redefined the MPP as the maximum empower principle (MEPP), i.e. all self-organizing systems tend to maximize their rate of emergy use or empower, and those systems that maximize empower will prevail in evolutionary competition^22^,^24^,^25^. Some theoretical and empirical research has supported the rationality of MEPP and showed its ability to be used to study the development of self-organizing systems^26^,^27^,^28^,^29^. Recent case studies confirmed that the development of subtropical forest plantations follow the MEPP. For example, all three forest plantations examined by South China Botanical Garden scientists from 1984 to 2007 progressively increased empower density (sej/ha/yr) for a variable number of years after planting^30^,^31^. After empower density peaked, it reached a dynamic equilibrium with fluctuations corresponding to variations of annual rainfall.

It follows from the MPP and it is generally accepted that ecosystems are constrained by evolutionary competition to maintain their structures and move them further away from thermodynamic equilibrium. Combined with Darwin’s survival of the fittest, the eco-exergy theory proposes that the available energy (exergy) of ecosystem components includes not only the free energy of matter but also the available energy of embodied information that they utilize to perform their life processes. Jørgensen & Mejer^23^,^32^ define eco-exergy as the available energy in units of kilojoules of all biotic components in an ecosystem compared with their surroundings, and they calculated it as the multiplication of biomass with available energy including the genetic information per unit biomass based on Kullbach’s measure of information of the genes, which was recently confirmed by using Boltzmann’s equation for the free energy of information^33^. The maximization of eco-exergy has been confirmed by several dynamic models of ecosystem structure primarily using aquatic^34^,^35^ and wetland^36^,^37^,^38^,^39^ case studies. The maximum eco-exergy principle states that ecosystems have the tendency to move away from thermodynamic equilibrium, losing structural entropy and gaining exergy and information in the process, i.e., ecosystems will develop towards higher levels of eco-exergy through the accumulation of chemical energy in organic matter, as well as maximizing the information embodied in the living organisms^40^. However, up to the present time, eco-exergy has been applied rarely to the analysis of forest systems^41^. In addition, we have not found any consideration of the applicability of the maximum eco-exergy principle to forest development in the published literature.

Eco-exergy is the reflection of the current stocks of available energy, while emergy can quantify the historical memory of development and maintenance in the system network. Therefore, the ratios of the eco-exergy to emergy (Ex/Em) and eco-exergy to empower (Ex/Emp) are informative indicators for the efficiency with which a system uses external energies to form and maintain the stability of its own thermodynamic structure and information content^41^,^42^,^43^,^44^. These indices have been recommended for the assessment of ecosystem health and also as a supplement to eco-exergy as a measure of sustainability^40^. Based on results from experimental phytoplankton microcosms^45^, Bastianoni *et al.*^46^ proposed that both the ‘maximum eco-exergy principle and the maximum empower principle are valid, but that there is an order in the maximizations, empower first and then eco-exergy. Bastianoni *et al.*^46^, also proposed that the eco-exergy/empower ratio should trend toward a maximum during ecosystem development, which has not been tested yet.

Based on over 20 years of monitoring at Heshan National Field Research Station of Forest Ecosystems, Guangdong providence, China^47^,^48^,^49^,^50^, the eco-exergy structural development of a mixed native species plantation (NP), a conifer plantation (CP) and an *Acacia mangium* plantation (AP) were quantified from 11 to 26 years old, by employing both the C-value method (CV) and the suggested weighting factors method (SWF) to calculate eco-exergy^51^,^52^,^53^,^54^,^55^,^56^,^57^,^58^,^59^,^60^. The efficiency of the development and maintenance of structure is measured through the two integrated eco-exergy and emergy indices mentioned above, i.e. the eco-exergy to emergy and eco-exergy to empower ratios, based on the simulation of the Biome-BGC model^30^, using the 9.26E + 24 sej/yr planetary emergy baseline^61^,^62^.

Special attention was paid to the following questions: (1) “Does the structural development of subtropical forest plantations follow the maximum eco-exergy principle, similar to wetlands^34^,^35^ and aquatic ecosystems^36^,^37^,^38^,^39^?”; (2) “Do structure and function develop synchronously?”; (3) “What are the trends in the efficiency of thermodynamic structural development and maintenance over the course of forest plantation development?”; (4) “What are the main contributors and limits to the thermodynamic structural development of subtropical forest plantations?”; (5) “Are further forest stand management practices needed to help subtropical forest plantations ‘jump over’ obstacles to their next succession stage?; If yes, when and what kind of management actions should be taken?”.

To answer the first and fourth questions, the thermodynamic structures of three subtropical forest plantations were quantified based on long-term monitoring and two calculation methods commonly applied, i.e. the suggested *β* value or SWF method and the C-value method. Then, combined with simulated results from the Biom-BGC model, the answers for the second and third questions were explored through the calculation of two integrated eco-exergy and emergy indices, i.e. eco-exergy/total emergy used in past years and eco-exergy/empower of the specific year (Table 1). Finally, some management suggestions were brought up, based on the combination of this study and other relative measurements, e.g., seed rain and the soil seed bank etc.

## Results

### Eco-exergy

**Figure 1:**
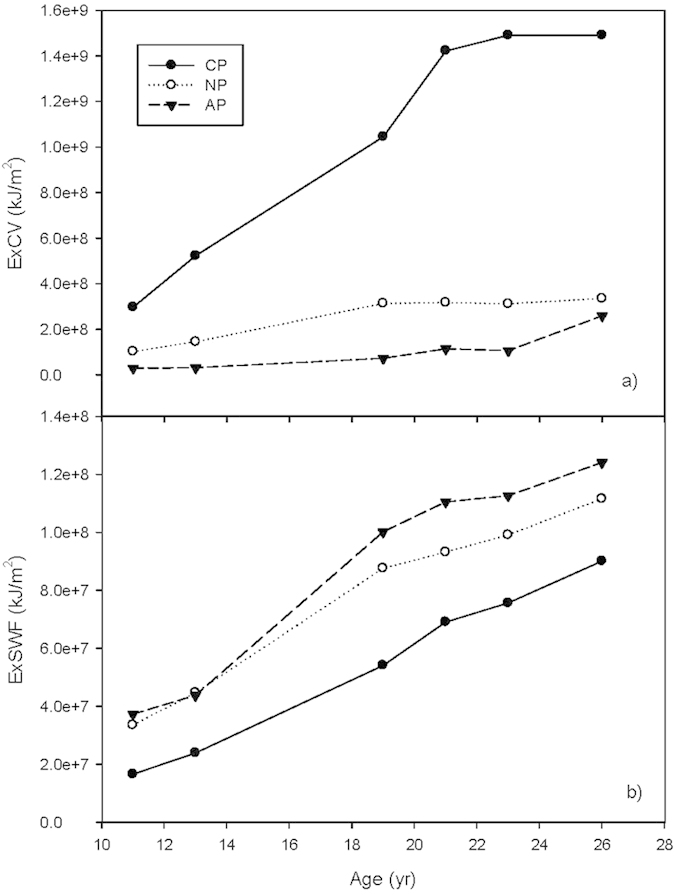
Eco-exergy of the three forest plantations. (**a**) Eco exergy based on the C-value weighting method (ExCV). (**b**) Eco-exergy based on the suggested weighting factors (ExSWF).

The eco-exergy of all three forest plantations increased over 3 times during the study period, no matter which weighting factor method was employed. However, the rate of eco-exergy accumulation slowed down in all 3 plantations after about 20 years of development, especially the eco-exergy as calculated using the C-value weighting method (ExCV, Fig. 1a). After 2 to 5 years of slower accumulation, the eco-exergy of all 3 plantations speeded up again, except that of the conifer plantation, CP.

The eco-exergies of CP, as measured by the C-Value method, were larger than that in the other two plantations (Fig. 1a), due to the high C-values of *Pinus massoniana* and *Cunninghamia lanceolata,* which have much higher C-values than most of the angiosperms found in the three plantations during the study period (Appendix A). Conversely, by using the suggested weighting factors method, CP was shown to have the lowest eco-exergy (ExSWF) over the study time (Fig. 1b), while AP had the highest eco-exergy. In contrast, AP had the lowest eco-exergy when measured using the C-value method (compare Fig. 1a,b).

### Biomass

**Figure 2:**
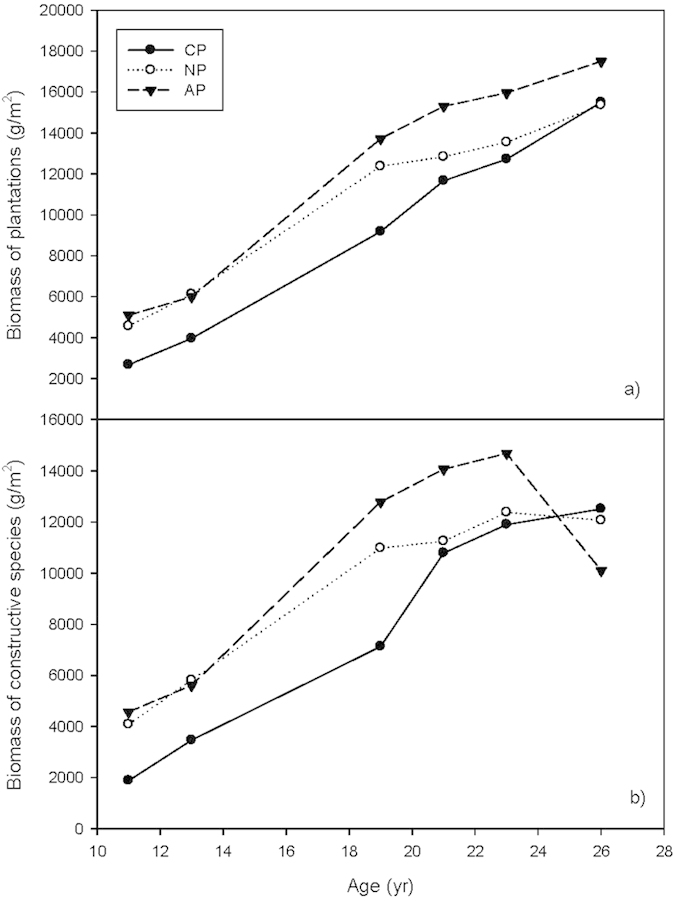
Biomass of three forest plantations. **(a)** Biomass of the whole plant communities. (**b**) Biomass of the original dominant species.

The biomass in all three plantations monotonically increased during the study period, with AP having the largest biomass at most times, followed by NP, and then CP (Fig. 2a). The rate of biomass increase begins to slow between 19 and 21 years for AP and NP, but this effect is not seen in CP until 21 to 23 years. After 2 to 5 years of slower growth, the rate of biomass accumulation sped up again, especially in CP, but also in AP and NP as well, even though the growth rate of the dominant species planted in AP (*Acacia mangium*) and NP (*Schima wallichii*) decreased (Fig. 2b). Biomass continued to accumulate in CP throughout the 26 years of observation and at this time it exceeded that of the native species plantation which was 1.7 times larger than CP when the plots were 11 years old (Fig. 2a).

During the first 23 years, the biomass accumulation in the *Acacia* plantation was mostly due to the growth of the dominant species, *Acacia mangium*, which experienced a rapid growth period for the first 20 years, and then slowed down. After 4 years of slower growth, the biomass of *Acacia mangium* decreased quickly after 23 years, but the biomass accumulation rate of the whole plant community continued to increase due to the growth of other species (Fig. 2a,b).

Like AP, NP experienced a rapid growth period in the first 20 years, followed by a 4-year period of leveling with no clear biomass accumulation, after which it again returned to more rapid growth, although the biomass of the dominant species planted, *Schima wallichii*, started to decrease (Fig. 2a,b).

Originally, the biomass in CP was lower than that in the other 2 plantations, but it maintained a near linear increase from 11 to 19 years old and increased its growth rate rapidly from 19 to 21 years, followed by slowing growth from 21 to 23 years, at a rate that was slightly lower than at other times in the period under study. Although the growth rate of the dominant species*, Pinus massoniana*, slowed down after 21 years, the biomass of the whole community continued to increase rapidly compared with the other two plantations (Fig. 2a,b). This result was seen because *Cunninghamia lanceolata* gradually took over the dominant place in the community. In addition, the understory community of broadleaf species within CP, e.g. *Schima superba, Schefflera octophylla*, and *Evodia lepta* etc. (Appendix B) grew rapidly during this time.

### Specific eco-exergy

**Figure 3:**
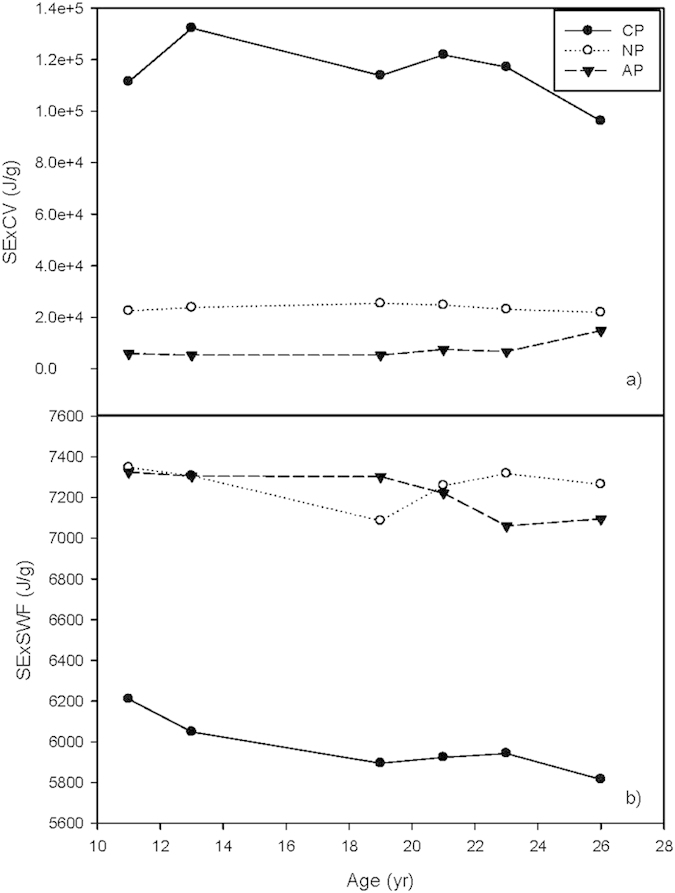
Specific eco-exergy of three forest plantations. (**a**) Specific eco-exergy based on the C-value weighting method (SpExCV). (**b**) Specific eco-exergy based on the suggested weighting factors method (SpExSWF).

Defined as the quantity of eco-exergy per unit biomass^54^,^63^, specific eco-exergy (SpEx) is another variable affecting eco-exergy besides biomass, which can be considered as a quality factor reflecting how developed the system is and to what extent biomass contributes to eco-exergy due to its content of information^40^,^54^. The results showed that different plantations showed different development dynamics of SpEx, which was also directly influenced by the weighting factor method used, e.g., CP had the highest specific eco-exergy based on the C-value method (SpExCV), but the lowest specific eco-exergy based on the suggested weighting factors method (SpExSWF) (Fig. 3a,b).

The specific eco-exergy in CP generally decreased during the study period, and the values based on the C-value weighting method (SpExCV) are over 15 times those based on SWF method (SpExSWF) (Fig. 3a,b). The reason behind the decrease of SpExCV is mainly because *Cunninghamia lanceolata* gradually took the place of *Pinus massoniana* as the main contributor to biomass accumulation in the plant community, and the C-value of *Cunninghamia lanceolata* is only 0.55 times that of *Pinus massoniana* (Appendix A,B).

The SpExCV of NP was about 3 times its SpExSWF, and the two estimates showed different trends with the latter decreasing before it was 19 years old and then increasing from 19 to 23 years old after which it decreased again, whereas the former remained relatively constant over the study period (Fig. 3a,b). Most of the specific eco-exergy in NP was contributed by *Schima wallichii*, and the community complexity did not fluctuate much during the study time (Appendix C,E).

The SpExCV of AP was lower than its SpExSWF during most of the study period. Both the SpExCV and SpExSWF of AP decreased slightly before the planting was 19 years old, followed by a 5-year perturbation, although the directions of the change in the specific eco-exergy were different with SpExSWF showing a decease and SpExCV an increase. In both cases, after 23 years, these perturbations were followed by a period of increasing specific eco-exergy (Fig. 3a,b).

### Development of eco-exergy and specific eco-exergy in different layers

**Figure 4:**
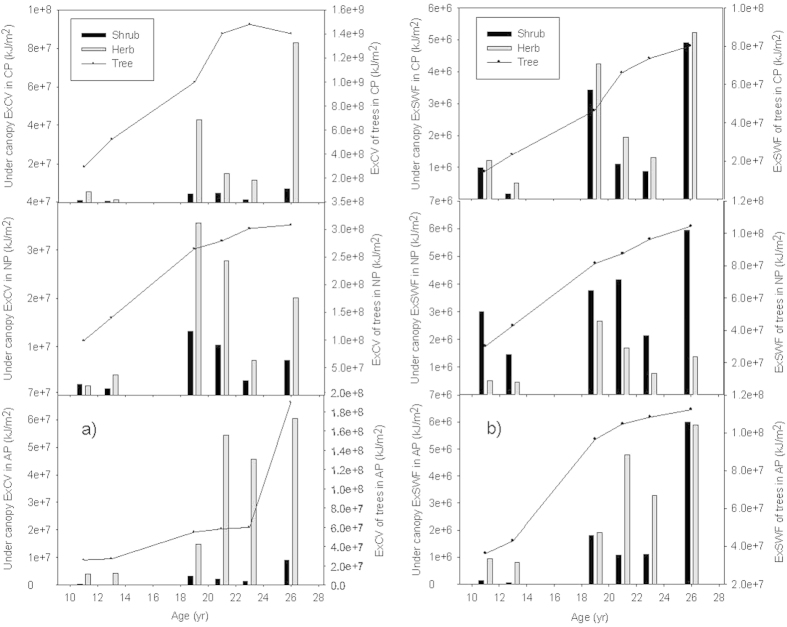
Eco-exergy development at different layers in the three forest plantations. **(a)** ExCV at different layers in the three forest plantations. (**b**) ExSWF at different layers in the three forest plantations.

Trees were the main contributors of eco-exergy in all 3 plantations over the whole study period, and their eco-exergy was at least an order of magnitude higher than that of the shrub and herb layers. Trees were followed by herbs and then shrubs in order from high to low eco-exergy, except the ExSWF of NP where the eco-exergy of shrubs exceeded herbs (Fig. 4a,b). The reasons that ExCV in the herb layer of NP was larger than that in the shrub layer but the ExSWF of the herb layer was smaller than that of the shrub layer is mainly due to the existence of *Blechnum orientale* in the herb layer which has a relatively low SWF, 158, but a high C-value, 10 (Appendix A,C).

The eco-exergy of tree, shrub and herb layers did not develop synchronously in all three plantations. The eco-exergy of trees continued to increase over most of the study period, but the rate of accumulation slowed down after 19 years, for example the ExCV of the trees in CP began to decrease after 23 years. In contrast, larger variations and unclear trends were seen in both the shrub and herb layers, although the eco-exergy of both layers in the 3 plantations was larger at 26 years than it was at 11 years (Fig. 4a,b).

**Figure 5:**
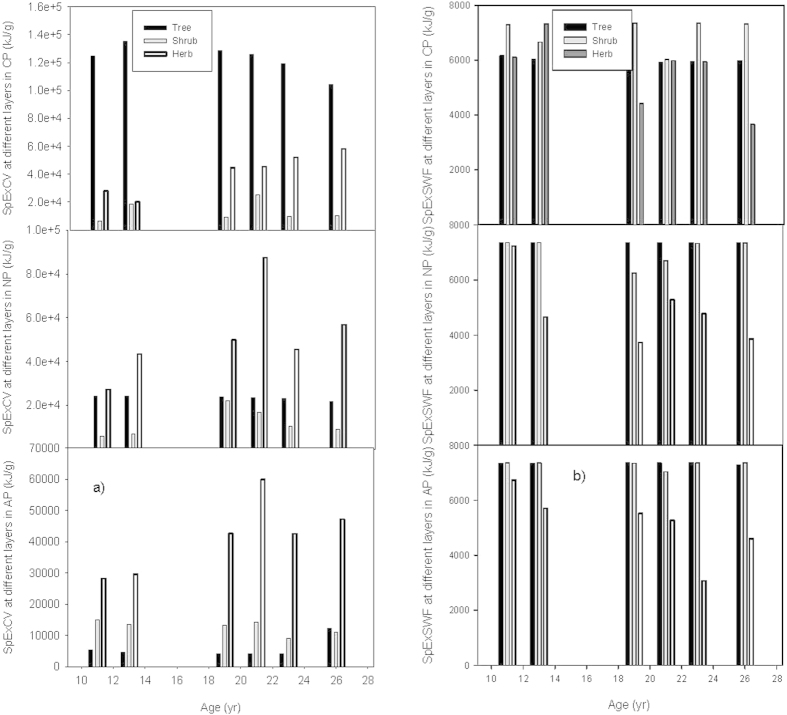
Specific eco-exergy development in different layers of the three forest plantations. (**a**) SpExCV of different layers in the three forest plantations. (**b**) SpExSWF of different layers in the three forest plantations.

The herb layer had higher SpExCVs in NP and in AP compared with that of the tree and shrub layers, but not in CP due to the high C-value of conifer trees. The SpExSWFs of the tree and shrub layers were higher than that of the herb layer in NP and AP, and at most times this is true for CP as well. Furthermore, the differences among SpExSWFs of the 3 layers were not large, compared with that among SpExCVs (Fig. 5a,b).

The development of specific eco-exergy showed no clear increasing trend during the study period, in the tree, shrub and herb layers, based on both of the two weighting methods.

### Thermodynamic efficiency in developing and maintaining structures

**Figure 6:**
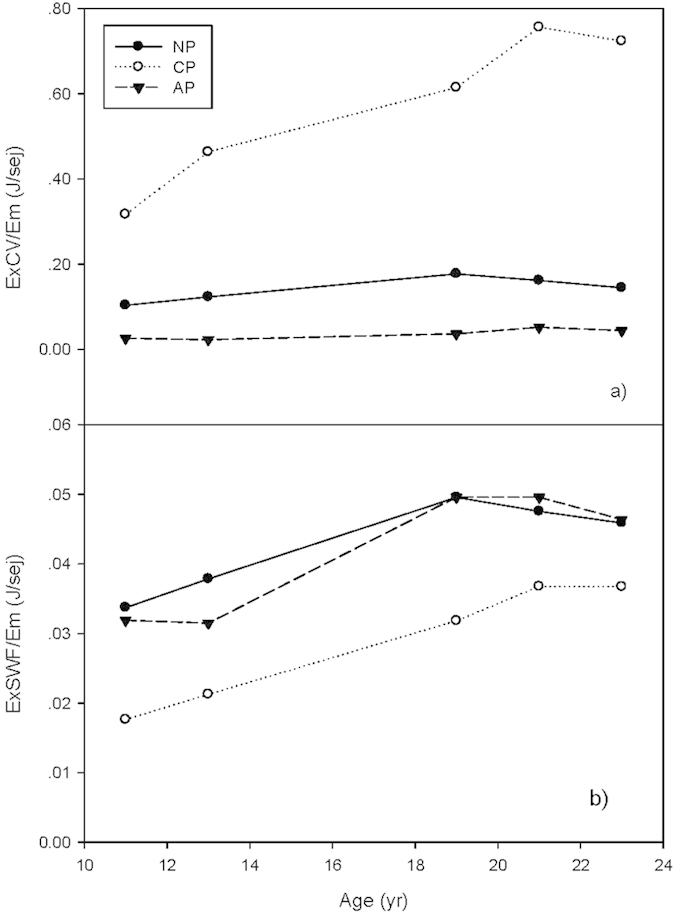
Eco-exergy to emergy ratios of the three plantations. **(a)** Eco-exergy to emergy ratios based on the CV method (ExCV/Em). (**b**) Eco-exergy to emergy ratios based on the SWF method (ExSWF/Em).

The trends in the eco-exergy to emergy ratios determined by both CV and SWF methods showed a rapid increase in the development efficiency of thermodynamic structure before year 19 in all three forest plantations. Then, the rate of increase slowed down for NP and AP as determined by the SWF method. After year 21 the estimates of thermodynamic efficiency of structural formation decreased across all sites as measured by both methods. At 23 years old, the ExCV/Em ratios of CP, NP and AP, respectively, were 2.29, 1.40 and 1.72 times that of their values when they were 11 years old, and their ExSWF/Em ratios were 2.08, 1.36 and 1.45 times that when they were 11 years old (Fig. 6a,b).

CP had the highest thermodynamic efficiency of structure formation among the three plantations as measured by the ExCV/Em ratio, followed by NP and then AP, but CP had the lowest efficiency when eco-exergy was measured by the SWF method. After 19 years, a different order in the thermodynamic efficiencies of structure formation was found among the three forest plantations with eco-exergy measured by the SWF method, i.e., AP exceeded NP as the most efficient system at this time. Also, the two different weighting methods for calculating eco-exergy exhibited a different order of the efficiency of thermodynamic structure formation among the three plantations examined, i.e., the order of efficiency from highest to lowest was CP > NP > AP based on the CV method, but NP > AP > CP up to 19 years and after that from AP > NP > CP based the SWF method (Fig. 6a,b).

**Figure 7:**
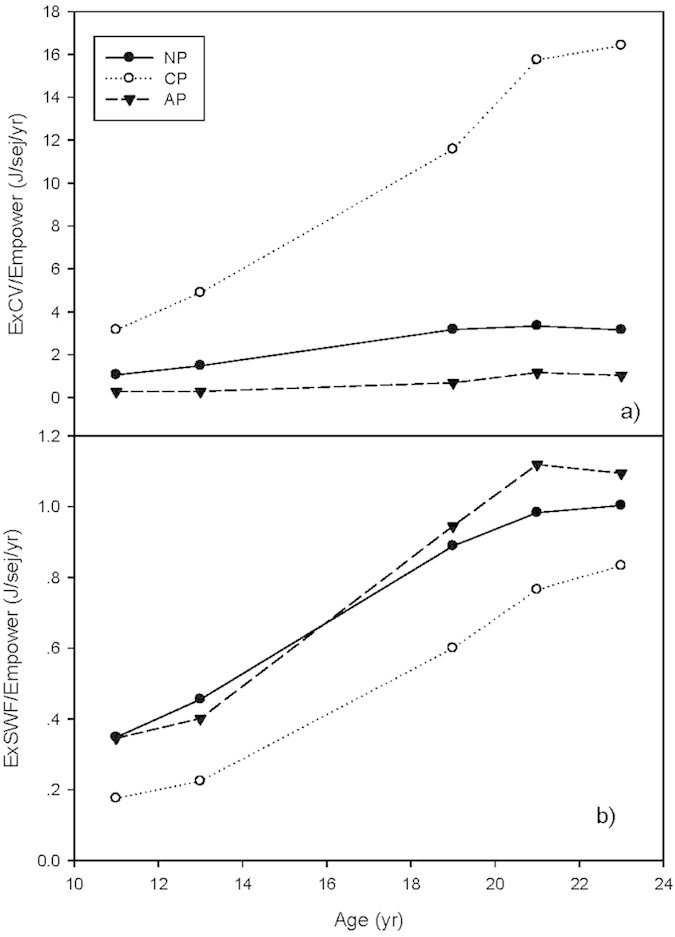
Eco-exergy to empower ratios of the three forest plantations. **(a)** Eco-exegy to empower ratios based on CV method (ExCV/Emp). (**b**) Eco-exergy to empower ratios based on SWF method (ExSWF/Emp).

The eco-exergy to empower ratio (Fig. 7a,b) showed that the maintenance efficiency for AP increased to a peak at 21 years old and then slowed down to a slight decrease, based on both the CV and SWF methods. The eco-exergy to empower ratio for CP increased more rapidly after 19 years and at a slower rate after 21 years of growth. The eco-exergy to empower ratio of NP peaked at 21 years and then slightly declined when measured by the CV method, whereas it began to slow its rate of growth progressively after 19 years when measured by the SWF method. At 23 years old, the ExCV/Emp ratios of CP, NP and AP were respectively 5.20, 2.97 and 3.74 times that when they were 11 years old; and the ExSWF/Emp ratios were 4.73, 2.88 and 3.17 times that when they were 11 years old (Fig. 7a,b).

Similar to the development efficiency (Fig. 6a), CP had the highest thermodynamic maintenance efficiency (Fig. 7a) among the three plantations according to the ExCV to empower ratio, followed by NP and then AP, but the lowest by the ExSWF to empower ratio (Fig. 7b). An inverse order of thermodynamic maintenance efficiency was shown among the three plantations depending on the weighting method used to determine eco-exergy, i.e., the order from highest to lowest maintenance efficiency went from CP to NP, and then to AP based on the C-value method, but the order was reversed after 19 years based the SWF method (Fig. 7a,b).

## Discussion

The eco-exergy of the three subtropical forest plantations increased over 3 times during the study period, as expected from the maximum eco-exergy principle, i.e. the development of the ecosystem always tends to enhance the thermodynamic structure by increasing its eco-exergy to move away from thermodynamic equilibrium^55^. No clear improvement in specific eco-exergy was observed for the three plantations during the study period. However, considering another aspect of structural change in the forest ecosystem, species richness (SR) increased in all three plantations over the study period (Appendix E). In the first 26 years of development, 38, 48 and 48 species were found in the study sites, CP, NP and AP, respectively. Thus, eco-exergy is an informative, but not a complete indicator for ecosystem development and self-organization, because it only covers two main aspects of development, i.e. biomass and genetic information, but not networks and the consequent emerging properties of ecosystems, which are the essential characteristics of systems, different from a simple combination of some isolated items^64^,^65^. How to account for eco-exergy in networks, in ecosystems and at multiple levels are some of the inevitable problems that need to be solved for further development of the eco-exergy methodology^66^,^67^.

The lack of synchronization in the development of structure (eco-exergy) and function (empower) in the three subtropical forest plantations was explored. After a rapid increase in empower during the first 3 to 5 years after planting, no further increase was observed in the three subtropical forest plantations from age 11 to 23 years old^30^, but eco-exergy increased over 3 times during this period. Consequently, an increasing trend in the eco-exergy to empower ratios was exhibited in all three forest plantations. This pattern supported the steady increasing efficiency trend proposed by Bastianoni *et al.*^46^ as the maximum eco-exergy to empower ratio, and explored by Tilley^29^ as maximum empower accompanied by minimum transformity. Both emergy and eco-exergy appear to be complementary concepts that may help uncover the general thermodynamic rules behind the complicated structural and functional dynamics of ecosystems, but the mechanisms governing their relationship have not been fully explored yet. Jørgensen *et al.*^68^ quantified the relationship between eco-exergy and emergy at the species level, and found that the emergy/eco-exergy ratios are between 3 and 400 times lower than the transformities, with the highest emergy to eco-exergy ratios corresponding to the most developed organisms. This gives us a reason to believe that the maximum eco-exergy to empower ratio have even clearer implications when ecosystems move toward higher stages of succession with more developed organisms taking the dominant place in the ecosystem, but specific simulation or case studies are still needed to demonstrate this hypothesis.

Trees were the largest contributors to the accumulation of eco-exergy in the three forest plantations, especially the dominant tree species planted initially. Consequently, the accumulation speed of eco-exergy slowed down after these dominant trees passed through their rapid growth periods around 20 years after planting. After a 2 to 5 year period of leveling, the growth of other species sped up the accumulation of eco-exergy in all three communities. Detailed results showed that most of these species were herbs, shrubs or small trees, e.g., *Blechnum orientale* and *Evodia lepta* in CP (Appendix B), *Pterospermum heterophyllum, Litsea cubeba* and *Blechnum orientale* in NP (Appendix C), and *Psychotria rubra, Toxicodendron succedaneum* and *Litsea glutinosa* in AP (Appendix D). The development of dominant large tree species for the next stage of succession has not yet occurred in any of these systems. Further measurements showed that the seeds supplied through seed rain and from the soil seed banks were dominated by shrub and herb species, especially *Cyrtococcum patterns*, while indigenous tree species were rare^69^,^70^. Thus, presently, both soil seed banks and the seed supplied from outside the system cannot drive succession of the three forest plantations to the natural climax stage. Introduction of suitable indigenous species to each of the plantations by artificial seeding or seedling planting should be considered to accelerate the succession of the plantations toward a more natural end-state^71^,^72^.

Both the suggested weighting factors (SWF) and the C-value (CV) weighting methods were applied in this study for comparison’s sake, considering that there are difficulties with both of these methods, e.g., only one *β* was given for each large organism group in the SWF method, so 158 was used for all ferns, 314 for all gymnosperms, and 393 for all angiosperms^53^. Also, the effect of the C-value paradox* has been shown to be a factor in the interpretation of eco-exergy calculated from the CV method^41^. Another difficulty with the CV method is that data on the amount of genetic material in all organisms is not currently available.

Our study showed that the application of these two methods to determine the eco-exergies in the same case study can result in very different and even opposite results, e.g., the ExCVs of CP were higher than those of NP and AP, but the ExSWFs of CP were lower than the other two plantations. However, each method also has advantages, e.g., a linear correlation was found between evolution time and the logarithm of the SWFs^57^. Also, the energy of information calculated by a new method based on Boltzmann’s equation gave approximately the same results as found from the SWFs method based on Kullbach’s measure previously given^33^. In addition, and the C-value does correlate with a range of features at the cellular and organismic levels, including cell size, cell division rate, and depending on the taxon, body size, metabolic rate, developmental rate, organ complexity, geographical distribution, and extinction risk^73^,^74^. Thus, the C-value may affect or be related to the adaptability or survivability of a species. The results of this study indicate that the integrated application of the two methods can be used to select tree species for forest restoration and management, e.g., species appearing earlier in evolution with large C-values, like *Pinus massoniana* might be ideal pioneer species for restoration, while highly indigenous species with large SWFs like *Castanopsis chinensis* might be ideal species to plant for furthering thermodynamic structural development^72^.

## Conclusions

The development of the three subtropical forest plantations followed the proposed maximum eco-exergy principle, as evidenced by the fact that the eco-exergy of all systems increased over 3 times during the study period.

The biomass and specific eco-exergy did not accumulate synchronously in the three subtropical forest plantations. The accumulation of eco-exergy was primarily driven by the increase in biomass during the study period. No clear increase in specific eco-exergy was observed for the three plantations, although the species richness did increase.

The structure (eco-exergy) and function (empower) did not develop synchronously in the subtropical forest plantations studied. The eco-exergy to total emergy and eco-exergy to empower ratios increased during the study period, but the rate of increase slowed down around 20 years after planting.

Trees are the largest contributors to the eco-exergy of the three plantations, especially the dominant trees planted initially. Introduction of suitable indigenous tree species to the plantations by artificial seeding or by planting seedlings should be considered after 20 years, since the seeds of indigenous tree species were rare in both the seed rain and in the soil seed banks.

Both C-values and suggested weighting factors have their limits and advantages for the determination of eco-exergy and its accumulation within a system. We showed that the application of both methods to characterize the same systems can result in different and even opposite results, but also that each method may give some useful information for forest management.

## Methods

### Site description

The study sites are located at the Heshan National Field Research Station of Forest Ecosystems, (112°53′15″–112°54′00′E, 22°40′07″–22°41′07″N), Heshan, Guangdong province, China. Under the control of a typical subtropical monsoon climate, the area has an annual average net solar radiation of about 4.6E + 9 J/m^2^, a 21.7 °C mean annual temperature, and an 1800 mm annual precipitation^26^,^37^. Subtropical monsoon evergreen broadleaf forest is the climax forest ecosystem and laterite is the zonal soil in this region. Guangdong is one of the most rapidly developing areas in China, and as a result it had about 3.74 million ha of degraded hillsides in the 1980s accompanied by serious environmental problems especially soil erosion and vegetation degradation^43^,^45^. In the 1980s, forest restoration was begun by the local government as one of the main strategies to promote regional sustainable development^44^. The three forest plantations under study, i.e., a mixed native broadleaf forest (NP, 2.68 ha), a mixed conifer forest (CP, 3.17 ha), and an *Acacia mangium* plantation (AP, 4.58 ha), were all planted in 1984 on grassy slopes covered by a few *Pinus massoniana* trees that were formed from the degradation of a regional monsoon evergreen broadleaf forest. The main species in NP is *Schima wallichii,* whereas, *P. massoniana* and *Cunninghamia lanceolata* dominate in CP. After the initial planting, ecosystem structure has developed gradually in all three plantations with regard to both plant biomass and species diversity (Appendix E).

### Community investigation and biomass measurement

Community survey plots of different sizes were set up for the investigation of changes in forest structure as follows: 100 m^2^ for trees, 25 m^2^ for shrubs, and 1 m^2^ for herbs. In the winter of 1995, 1997, 2003, 2005, 2007 and 2010, the following measurements were made in the survey plots: the diameter at breast height (DBH), height (H) and canopy area (CA) of all the trees; the stem diameter, height and canopy area of shrubs and tree seedlings under 2 m tall, and the height and coverage of herbs. Eight trees and 10 shrubs with characteristics close the average H, DBH and CA for each species were harvested from nearby the survey plots to determine the biomass equations for the trees, and the three dominant shrub species^46^. Then, the biomasses of trees and shrubs were calculated based on the empirically determined biomass relationships for the main species. To represent the general condition of herbs in each plantation, three 1 m^2^ quadrats of the herbs with near average height and coverage were harvested for the measurement of biomass. All biomass, eco-exergy and empower values in the three forest plantations were calculated on the basis of 1 ha.

### Calculation of eco-exergy and specific eco-exergy

The eco-exergy of each community was computed using Eq. (1):


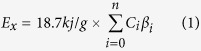


where 18.7 kJ/g is the mean eco-exergy of detritus or dead organic matter; *C*_*i*_is the biomass of the ith species (g/m^2^) and *β*_*i*_is the weighting factor of the genetic information quantified by estimating the chemical potential energy of the *i*th species in the system relative to the chemical potential energy of biomass as detritus^51^, which is taken as a measure of the information embodied in the living organisms in the system compared to the chemical energy in dead organic matter. Thus *β* is an estimate of the distance of the living matter in the ecosystem from thermodynamic background assumed to be dead organic matter. It is assumed to be proportional to the available energy or “exergy” in the living components of an ecosystem.

There are two methods commonly applied to calculate eco-exergy, i.e. directly using the suggested *β* value (SWF) based on Kullbach’s measurement of information, i.e. genome size and the complexity of different organisms^40^,^51^,^57^, and the *β* value calculated from the C-value, which is the amount of DNA contained within a haploid nucleus as measured by the number of base pairs (bp). Using the C-value method, the weighting factor *β* is calculated through the following equation:


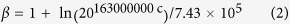


where 7.43 × 10^5^ is the contribution of detritus to the eco-exergy in g/l; *c* is the total quantity of DNA in the diploid genome of the plant cell in pico grams (pg) where 1 pg = 0.98 × 10^9^ base pairs (bp). So, c in pg is multiplied by 1.63 × 10^8^ is the number of nucleotide triplets. The number of nucleotide triplets is the maximum coding capacity of the haploid genome, because each adjacent triplet of nucleotides corresponds to a transcribed RNA-signal^59^,^60^. Twenty is the number of possible amino acids that can be coded by each triplet. All C-values used in this study were obtained from the plant DNA C-values Database of the Royal Botanic Gardens, Kew, UK ( http://data.kew.org/cvalues). For those species not in that database, the mean C-value for the genus or the family to which they belonged was used. Three suggested weighting factors, i.e., 158 for ferns, 314 for gymnosperms, and 393 for angiosperms, were used to represent the three plant communities analyzed in this study^52^,^53^,^54^,^55^,^56^.

Specific Eco-exergy was calculated using Eq. (2):


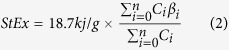


where all factors and variables (*C*_*i*_and *β*_*i*_) are defined in the same manner as those in Eq. (1).

### Measurement of the thermodynamic efficiency in developing and maintaining ecosystem structures

The ratio of eco-exergy to total emergy used in the past years to develop the system structure was calculated to evaluate the thermodynamic efficiency of structure formation, while the ratio of eco-exergy to empower of the system in a specific year was employed to measure the thermodynamic efficiency of the system in maintaining that structure. Empower was defined as the flow of emergy per unit time^24^, in sej/ha/yr and in this study it was obtained from a forest ecosystem network simulation based on the Biome-BGC model^26^ using the 9.26E + 24 sej/yr planetary emergy baseline^61^,^62^.

## Additional Information

**How to cite this article**: Lu, H. *et al.* Eco-exergy and emergy based self-organization of three forest plantations in lower subtropical China. *Sci. Rep.* **5**, 15047; doi: 10.1038/srep15047 (2015).

## Supplementary Material

Supplementary Information

## Figures and Tables

**Table 1:** Eco-exergy and emergy indices used in this study.

**Index**	**Description**
Eco-exergy	The available energy of all living biotic components in an ecosystem compared to the nonliving state, i.e., detritus, in units of kilojoules, which can be used to measure the degree of thermodynamic organization and health of the system^32^.
Specific eco-exergy	The quantity of eco-exergy per unit biomass of a community, in units of kilojoules/g, which can be used as a quality factor reflecting how developed the system is due to its content of genetic information^32^.
Emergy	The available energy of one kind previously used up directly and indirectly to make a service or product, generally in units of solar emjoules (sej), which is a donor method of valuation^24^.
Empower	The quantity of emergy flowing through a system per unit time, which is a measure of the success of resource capture and the degree of development of a system^24^.
Eco-exergy/emergy	The quantity of eco-exergy built up in systems per unit of the emergy stored over time, which is a measure of the thermodynamic efficiency of the ecosystem in building structure^41^.
Eco-exergy/empower	The quantity of eco-exergy contained in systems per unit of emergy flowing in the system, which is a measure of the thermodynamic efficiency of ecosystem in maintaining structures^41^.
