# Supplementary material for: Eco-exergy and emergy based self-organization of three forest plantations in lower subtropical China
Source: Sci Rep. 2015 Oct 21;5:15047. doi: 10.1038/srep15047 (PMC4613664; doi:10.1038/srep15047)
Supplement: Supplementary Information [file srep15047-s1.doc]

**Eco-exergy and emergy based self-organization of three forest plantations in lower subtropical China**

Hongfang Lu^1^, Fangyan Fu^1^, Hao Li^1^, Daniel E. Campbell^2^, Hai Ren^1*^

^1^Key Laboratory of Vegetation Restoration and Management of Degraded Ecosystems, South China Botanical Garden, Chinese Academy of Sciences, Guangzhou 510650, China

^2^US EPA, Office of Research and Development, National Health and Environmental Effects Research Laboratory, Atlantic Ecology Division, 27 Tarzwell Drive, Narragansett, RI, USA

Appendix A. C-values and SWFs applied to each species in the three subtropical forest plantations under study

| No. | **Latin Name** | **SWF*** | **C value**** | **No.** | **Latin Name** | **SWF*** | **C value**** |
| --- | --- | --- | --- | --- | --- | --- | --- |
| 1 | *Smilax china* | 393 | 5 | 54 | *Toxicodendron vernicifluum* | 393 | 2.37 |
| 2 | *Dianella ensifolia* | 393 | 41.81 | 55 | *Toxicodendron succedaneum* | 393 | 2.37 |
| 3 | *Cibotium barometz* | 158 | 7.18 | 56 | *Lagerstroemia* | 393 | 0.99 |
| 4 | *Schima superba* | 393 | 3.9 | 57 | *Morinda parvifolia* | 393 | 0.65 |
| 5 | *Schima Wallichii* | 393 | 3.9 | 58 | *Gardenia jasminoides* | 393 | 1.3 |
| 6 | *Hyptis suaveolens* | 393 | 2.9 | 59 | *Psychotria rubra* | 393 | 1.03 |
| 7 | *Mallotus apelta* | 393 | 3.82 | 60 | *Spermacoce latifolia* | 393 | 1.07 |
| 8 | *Mallotus paniculatus* | 393 | 3.82 | 61 | *Geum aleppicum* | 393 | 1.07 |
| 9 | *Breynia fruticosa* | 393 | 3.82 | 62 | *Mussaenda pubescens* | 393 | 1.07 |
| 10 | *Glochidion eriocarpum* | 393 | 3.82 | 63 | *Rhaphiolepis indica* | 393 | 0.8 |
| 11 | *Sapium discolor* | 393 | 3.82 | 64 | *Rubus alceaefolius* | 393 | 0.37 |
| 12 | *Glochidion puberum* | 393 | 3.82 | 65 | *Cotoneaster horizontalis* | 393 | 2.86 |
| 13 | *Bridelia monoica* | 393 | 1.25 | 66 | *Rosa cymosa* | 393 | 0.73 |
| 14 | *Phyllanthus urinaria* | 393 | 1 | 67 | *Radix Wikstroemae* | 393 | 2.29 |
| 15 | *Aporosa dioica* | 393 | 3.82 | 68 | *Centella asiatica* | 393 | 2.42 |
| 16 | *Ilex asprella* | 393 | 1.47 | 69 | *Ficus variolosa* | 393 | 0.71 |
| 17 | *Ilex triflora* | 393 | 1.47 | 70 | *Ficus irisana* | 393 | 0.71 |
| 18 | *llex franchetiana* | 393 | 1.47 | 71 | *Ficus hirta* | 393 | 0.71 |
| 19 | *Desmos chinensis* | 393 | 1.2 | 72 | *Cyperus rotundus* | 393 | 0.45 |
| 20 | *Pteris semipinnata* | 158 | 5.97 | 73 | *Camellia sinensis* | 393 | 3.9 |
| 21 | *Pteris multifida* | 158 | 5.97 | 74 | *Eurya chinensis* | 393 | 3.9 |
| 22 | *Pteris ensiformis* | 158 | 5.97 | 75 | *Cunninghamia lanceolata* | 314 | 14.17 |
| 23 | *Acacia mangium* | 393 | 0.65 | 76 | *Nephrolepiscordifolia* | 158 | 8.63 |
| 24 | *Lophatherum gracile* | 393 | 7.76 | 77 | *Dioscorea bulbifera* | 393 | 1.4 |
| 25 | *Eragrostis atrovirens* | 393 | 0.53 | 78 | *Pinus massoniana* | 314 | 25.79 |
| 26 | *Panicum brevifolium* | 393 | 1.65 | 79 | *Pinus elliottii* | 314 | 23.3 |
| 27 | *Cyrtococcum patens* | 393 | 7.76 | 80 | *Baeckea frutescens* | 393 | 0.75 |
| 28 | *Setaira viridis* | 393 | 7.76 | 81 | *Syzygium hainanense* | 393 | 1.83 |
| 29 | *Miscanthus sinensis* | 393 | 7.76 | 82 | *Syzygium rehderianum* | 393 | 1.83 |
| 30 | *Paspalum distichum* | 393 | 1.09 | 83 | *Syzygium jambos* | 393 | 1.83 |
| 31 | *Ishaemum indicum* | 393 | 7.76 | 84 | *Rhodomyrtus tomentosa* | 393 | 0.75 |
| 32 | *Ottochloa nodosa* | 393 | 7.76 | 85 | *Adiantum flabellulatum* | 158 | 7.67 |
| 33 | *Cratoxylon ligustrinum* | 393 | 0.4 | 86 | *Nvicrolepia hancei* | 158 | 7.94 |
| 34 | *Cyclosorus parasiticus* | 158 | 10 | 87 | *Blechnum orientale* | 158 | 10 |
| 35 | *Urena lobata* | 393 | 1.65 | 88 | *Pterospermum heterophyllum* | 393 | 0.8 |
| 36 | *HerbaElephantopi* | 393 | 3.74 | 89 | *Helicteres angustifolia* | 393 | 1.32 |
| 37 | *Eupatorium chinense* | 393 | 8.13 | 90 | *Aralia decaisneana* | 393 | 2.65 |
| 38 | *Herba Asteris* | 393 | 4.32 | 91 | *Schefflera octophylla* | 393 | 2.65 |
| 39 | *Castanopsis hystrix* | 393 | 0.86 | 92 | *Torenia concolor* | 393 | 0.17 |
| 40 | *Castanopsis fissa* | 393 | 0.86 | 93 | *Torenia glabra* | 393 | 0.17 |
| 41 | *Cymbidium* | 393 | 4.05 | 94 | *Melastoma dodecandrum* | 393 | 0.19 |
| 42 | *Dicranopteris dichotoma* | 158 | 6.4 | 95 | *Melastoma candidum* | 393 | 0.19 |
| 43 | *Melia azedarach* | 393 | 1.36 | 96 | *Trema orientalis* | 393 | 1.31 |
| 44 | *Aglaia odorata* | 393 | 0.55 | 97 | *Tetradium glabrifolium* | 393 | 1.36 |
| 45 | *Polygonum chinense* | 393 | 0.62 | 98 | *Zanthoxylum nitidum* | 393 | 6.15 |
| 46 | *Podocarpus nagi* | 314 | 9.9 | 99 | *Evodia lepta* | 393 | 1.36 |
| 47 | *Clerodendron fortunatum* | 393 | 1.34 | 100 | *Litsea rotundifolia* | 393 | 1.71 |
| 48 | *Michelia macclurel* | 393 | 4.04 | 101 | *Litsea glutinosa* | 393 | 2.82 |
| 49 | *Jasminum amplexicaule* | 393 | 1.44 | 102 | *Litsea cubeba* | 393 | 2.82 |
| 50 | *Ligustrum sinense* | 393 | 1.08 | 103 | *Lindera communis* | 393 | 1.71 |
| 51 | *Ligustrum quihoui* | 393 | 1.08 | 104 | *Cinnamomum burmanni* | 393 | 0.6 |
| 52 | *Vitis piasezkii* | 393 | 0.49 | 105 | *Cinnamomum camphora* | 393 | 0.6 |
| 53 | *Acer fabri* | 393 | 0.78 | 106 | *Embelia laeta* | 393 | 2.65 |

* Jørgensen, 2006a

** <http://data.kew.org/cvalues>, in pg.

Appendix B. Biomass composition in the conifer plantation (CP) during the study period (g/m^2^)

| Latin Name | Age | | | | | |
| --- | --- | --- | --- | --- | --- | --- |
|  | 11 | 13 | 19 | 21 | 23 | 26 |
| **Trees** |  |  |  |  |  |  |
| *Mallotus apelta* | 5.51 | 0.19 | 0.00 | 0.00 | 0.00 | 0.07 |
| *Mallotus paniculatus* | 0.00 | 6.46 | 0.00 | 0.00 | 0.00 | 0.00 |
| *Rhaphiolepis indica* | 0.29 | 0.21 | 0.00 | 0.00 | 0.00 | 0.00 |
| *Ficus hirta* | 0.00 | 0.00 | 0.00 | 3.36 | 0.00 | 0.49 |
| *Baeckea frutescens* | 0.47 | 0.00 | 0.00 | 0.00 | 0.00 | 0.00 |
| *Clerodendron fortunatum* | 0.39 | 0.29 | 0.00 | 0.00 | 0.00 | 1.10 |
| *Schima superba* | 211.79 | 327.01 | 618.03 | 296.85 | 191.33 | 526.70 |
| *Gardenia jasminoides* | 0.07 | 0.15 | 0.00 | 0.00 | 0.00 | 0.00 |
| *Litsea glutinosa* | 0.00 | 0.00 | 0.00 | 36.99 | 0.00 | 0.00 |
| *Psychotria rubra* | 0.00 | 0.00 | 0.00 | 0.00 | 0.00 | 1.34 |
| *Castanopsis fissa* | 0.00 | 0.00 | 0.00 | 0.00 | 2.44 | 1.33 |
| *Radix Wikstroemae* | 0.09 | 0.00 | 0.00 | 0.00 | 0.00 | 0.00 |
| *Pinus massoniana* | 1639.00 | 2852.76 | 5032.58 | 6322.01 | 6018.73 | 4071.74 |
| *Ilex asprella* | 41.07 | 31.55 | 0.00 | 0.00 | 0.00 | 5.73 |
| *Eurya chinensis* | 1.33 | 0.84 | 0.00 | 0.00 | 0.00 | 0.37 |
| *Evodia lepta* | 25.87 | 0.62 | 2.99 | 8.41 | 89.75 | 158.41 |
| *Litsea cubeba* | 0.00 | 0.00 | 0.00 | 12.61 | 50.73 | 3.52 |
| *Trema orientalis* | 0.00 | 0.00 | 0.00 | 0.00 | 0.00 | 0.75 |
| *Helicteres angustifolia* | 0.01 | 0.00 | 0.00 | 0.00 | 0.00 | 0.00 |
| *Cunninghamia lanceolata* | 197.40 | 509.54 | 2098.18 | 4462.53 | 5881.59 | 8435.82 |
| *pinus elliottii* | 48.96 | 107.72 | 0.00 | 0.00 | 0.00 | 0.00 |
| *Glochidion puberum* | 0.17 | 0.00 | 0.00 | 0.00 | 0.00 | 0.00 |
| *Rhodomyrtus tomentosa* | 162.77 | 26.02 | 0.00 | 0.00 | 0.00 | 0.00 |
| *Schefflera octophylla* | 0.00 | 0.00 | 0.00 | 10.65 | 140.85 | 161.36 |
| *Toxicodendron succedaneum* | 0.00 | 0.00 | 0.00 | 0.00 | 2.22 | 0.95 |
| *Cinnamomum burmanni* | 0.00 | 0.00 | 0.00 | 0.00 | 10.26 | 0.21 |
| *Michelia macclurel* | 0.00 | 0.00 | 0.00 | 0.00 | 0.00 | 32.07 |
| **Subtotal** | 2335.19 | 3863.36 | 7751.78 | 11153.41 | 12387.88 | 13401.96 |
|  |  |  |  |  |  |  |
| **Shrubs** | 0.00 | 0.00 | 0.00 | 0.00 | 0.00 | 0.00 |
| *Mallotus apelta* | 0.00 | 0.00 | 0.01 | 0.17 | 0.00 | 9.55 |
| *Mallotus paniculatus* | 0.00 | 1.29 | 0.00 | 0.00 | 1.62 | 0.00 |
| *Ficus variolosa* | 0.00 | 0.00 | 0.01 | 0.38 | 0.00 | 2.12 |
| *Litsea rotundifolia* | 0.00 | 0.00 | 0.00 | 0.00 | 0.26 | 0.44 |
| *Rhaphiolepis indica* | 0.00 | 0.00 | 1.22 | 0.76 | 0.89 | 6.52 |
| *Ficus hirta* | 0.00 | 0.00 | 0.00 | 0.58 | 4.72 | 7.60 |
| *Rubus alceaefolius* | 77.64 | 0.12 | 0.00 | 0.00 | 0.00 | 0.00 |
| *Baeckea frutescens* | 1.03 | 0.00 | 0.00 | 0.00 | 0.00 | 0.00 |
| *Clerodendron fortunatum* | 6.51 | 9.17 | 5.66 | 18.45 | 16.86 | 101.13 |
| *Breynia fruticosa* | 0.38 | 0.00 | 0.08 | 0.04 | 0.84 | 1.20 |
| *Eupatorium chinense* | 0.08 | 0.00 | 0.00 | 0.00 | 0.00 | 0.00 |
| *Gardenia jasminoides* | 1.35 | 1.19 | 0.60 | 2.35 | 4.87 | 12.96 |
| *Psychotria rubra* | 0.00 | 0.00 | 0.33 | 3.10 | 5.45 | 62.61 |
| *Zanthoxylum nitidum* | 0.00 | 0.00 | 0.00 | 0.03 | 0.00 | 0.00 |
| *Radix Wikstroemae* | 0.14 | 0.00 | 0.00 | 0.00 | 0.00 | 0.00 |
| *Glochidion eriocarpum* | 0.00 | 0.00 | 0.02 | 0.00 | 0.47 | 0.44 |
| *Ilex asprella* | 0.00 | 2.71 | 452.49 | 73.40 | 48.29 | 114.24 |
| *Eurya chinensis* | 1.95 | 2.69 | 0.43 | 2.39 | 3.58 | 22.60 |
| *Cotoneaster horizontalis* | 17.14 | 0.00 | 0.00 | 0.00 | 0.00 | 0.00 |
| *Toxicodendron vernicifluum* | 0.00 | 0.00 | 0.00 | 0.74 | 0.00 | 0.00 |
| *Evodia lepta* | 0.74 | 1.42 | 2.69 | 20.36 | 16.17 | 217.39 |
| *Litsea cubeba* | 0.00 | 0.00 | 2.56 | 1.35 | 3.71 | 18.67 |
| *Trema orientalis* | 0.00 | 0.00 | 0.00 | 0.00 | 2.77 | 0.00 |
| *Sapium discolor* | 0.00 | 0.00 | 0.00 | 0.00 | 1.83 | 1.25 |
| *Helicteres angustifolia* | 0.00 | 0.00 | 0.00 | 0.00 | 0.28 | 0.00 |
| *Ligustrumsinense* | 0.00 | 0.00 | 0.00 | 0.06 | 0.00 | 0.00 |
| *Cunninghamialanceolata* | 0.00 | 0.00 | 0.00 | 0.14 | 0.00 | 12.40 |
| *Embelia laeta* | 0.41 | 0.00 | 0.00 | 0.00 | 0.00 | 0.00 |
| *Glochidion puberum* | 0.29 | 0.00 | 0.01 | 0.04 | 0.00 | 0.00 |
| *Rhodomyrtus tomentosa* | 4.14 | 2.14 | 0.21 | 0.02 | 0.00 | 0.00 |
| *Bridelia monoica* | 0.00 | 0.00 | 0.07 | 0.47 | 0.00 | 8.61 |
| *Blechnum orientale* | 1.96 | 4.11 | 0.00 | 55.72 | 0.00 | 0.00 |
| *Lindera communis* | 0.00 | 0.00 | 0.00 | 0.00 | 0.00 | 2.81 |
| *Rosa cymosa* | 20.37 | 0.00 | 0.00 | 0.00 | 0.00 | 0.00 |
| *Ligustrum quihoui* | 0.00 | 0.00 | 0.00 | 0.00 | 0.00 | 3.37 |
| *Urena lobata* | 2.46 | 0.29 | 0.09 | 0.04 | 0.89 | 0.00 |
| *Schefflera octophylla* | 0.00 | 0.00 | 0.26 | 0.57 | 0.15 | 6.58 |
| *Melastoma candidum* | 0.00 | 0.95 | 0.00 | 0.00 | 1.06 | 15.98 |
| *llex franchetiana* | 0.00 | 0.00 | 0.13 | 0.00 | 0.00 | 0.00 |
| *Toxicodendron succedaneum* | 0.17 | 0.00 | 0.04 | 2.59 | 1.11 | 7.11 |
| *Phyllanthus urinaria* | 0.00 | 0.00 | 0.00 | 0.00 | 0.00 | 0.37 |
| *Cinnamomum burmanni* | 0.00 | 0.00 | 0.05 | 0.45 | 1.94 | 32.62 |
| *Cinnamomum camphora* | 0.00 | 0.00 | 0.00 | 0.04 | 0.00 | 0.00 |
| **Subtotal** | 136.75 | 26.07 | 466.93 | 184.25 | 117.75 | 668.57 |
|  |  |  |  |  |  |  |
| **Herbs** | 0.00 | 0.00 | 0.00 | 0.00 | 0.00 | 0.00 |
| *Pteris semipinnata* | 0.00 | 0.00 | 0.00 | 0.00 | 0.00 | 3.54 |
| *Ficus irisana* | 0.00 | 0.00 | 1.68 | 0.00 | 0.00 | 0.00 |
| *Lophatherum gracile* | 0.00 | 0.00 | 0.00 | 0.00 | 20.37 | 128.06 |
| *Melastoma dodecandrum* | 46.14 | 0.20 | 10.99 | 2.80 | 0.01 | 0.00 |
| *Pteris multifida* | 0.00 | 0.37 | 0.00 | 0.00 | 0.00 | 0.00 |
| *Baeckea frutescens* | 0.07 | 0.00 | 0.00 | 0.00 | 0.00 | 0.00 |
| *Cyrtococcum patens* | 1.76 | 13.46 | 0.00 | 0.00 | 0.00 | 0.00 |
| *Setaira viridis* | 0.00 | 2.20 | 0.00 | 0.00 | 0.00 | 0.00 |
| *Torenia glabra* | 0.00 | 0.00 | 2.14 | 0.00 | 0.00 | 0.00 |
| *Clerodendron fortunatum* | 5.16 | 21.97 | 4.71 | 17.23 | 0.00 | 0.00 |
| *Nvicrolepia hancei* | 0.00 | 0.00 | 115.36 | 3.64 | 9.91 | 208.69 |
| *Cyclosorus parasiticus* | 0.00 | 0.00 | 0.00 | 0.00 | 3.23 | 0.00 |
| *Eupatorium chinense* | 4.40 | 0.00 | 0.00 | 0.00 | 0.00 | 0.00 |
| *Gardenia jasminoides* | 0.00 | 0.00 | 3.05 | 0.00 | 0.00 | 0.00 |
| *Polygonum chinense* | 0.00 | 0.00 | 0.00 | 0.00 | 2.47 | 0.00 |
| *Cibotium barometz* | 0.00 | 0.00 | 326.86 | 0.00 | 0.00 | 0.00 |
| *Dicranopteris dichotoma* | 56.44 | 0.00 | 0.00 | 6.21 | 8.83 | 13.06 |
| *Ilex asprella* | 0.00 | 1.46 | 20.60 | 0.00 | 0.00 | 0.00 |
| *Jasminum amplexicaule* | 0.00 | 0.00 | 144.82 | 0.00 | 0.00 | 0.00 |
| *Evodia lepta* | 0.00 | 0.18 | 4.70 | 33.50 | 0.00 | 0.00 |
| *Cyperus rotundus* | 0.00 | 0.00 | 0.00 | 2.39 | 0.00 | 0.00 |
| *Herba Asteris* | 2.19 | 0.00 | 0.00 | 0.00 | 0.00 | 0.00 |
| *Dianella ensifolia* | 0.00 | 0.00 | 34.61 | 3.84 | 2.86 | 6.10 |
| *Adiantum flabellulatum* | 0.00 | 0.00 | 2.77 | 16.68 | 12.72 | 2.48 |
| *Vitis piasezkii* | 0.00 | 0.00 | 0.93 | 0.00 | 0.00 | 0.00 |
| *Embelia laeta* | 0.00 | 24.32 | 3.26 | 0.00 | 0.00 | 0.00 |
| *Glochidion puberum* | 19.78 | 0.00 | 0.00 | 0.00 | 0.00 | 0.00 |
| *Rhodomyrtus tomentosa* | 13.18 | 0.00 | 0.00 | 0.00 | 0.00 | 0.00 |
| *Lindsaea orbiculata* | 0.00 | 0.00 | 12.05 | 0.00 | 0.00 | 3.48 |
| *Blechnum orientale* | 0.00 | 0.00 | 166.12 | 69.22 | 36.89 | 967.70 |
| *Ishaemum indicum* | 48.39 | 0.00 | 0.00 | 0.00 | 0.00 | 0.00 |
| *Ottochloa nodosa* | 0.00 | 0.00 | 24.64 | 165.48 | 124.99 | 93.51 |
| *Urena lobata* | 0.01 | 0.00 | 62.57 | 0.00 | 0.00 | 0.00 |
| *Lindsaea heterophylla* | 0.00 | 0.00 | 17.33 | 5.83 | 0.34 | 0.00 |
| *Mussaenda pubescens* | 0.88 | 4.39 | 0.90 | 0.00 | 0.00 | 0.00 |
| **Subtotal** | 198.40 | 68.56 | 960.09 | 326.82 | 222.63 | 1426.62 |
|  |  |  |  |  |  |  |
| **Total** | 2670.34 | 3957.98 | 9178.79 | 11664.49 | 12728.26 | 15497.15 |

Appendix C. Biomass composition in the mixed native plantation (NP) during the study period (g/m^2^)

|  | Age | | | | | |
| --- | --- | --- | --- | --- | --- | --- |
| Latin Name | 11 | 13 | 19 | 21 | 23 | 26 |
| **Trees** |  |  |  |  |  |  |
| *Alangium chinense* | 0.00 | 0.00 | 0.00 | 17.85 | 23.82 | 0.00 |
| *Ficus variolosa* | 0.00 | 0.00 | 0.00 | 0.00 | 0.02 | 0.00 |
| *Litsea rotundifolia* | 0.10 | 0.11 | 0.00 | 7.93 | 2.03 | 1.28 |
| *Rhaphiolepis indica* | 0.00 | 0.00 | 0.00 | 0.00 | 0.00 | 0.21 |
| *Ficus hirta* | 0.00 | 0.00 | 0.00 | 9.91 | 0.00 | 0.25 |
| *Pterospermum heterophyllum* | 0.00 | 0.00 | 0.00 | 126.08 | 460.87 | 878.57 |
| *Clerodendron fortunatum* | 0.00 | 0.00 | 0.30 | 0.00 | 0.20 | 2.87 |
| *Schima superba* | 0.00 | 0.00 | 0.00 | 267.78 | 20.03 | 5.47 |
| *Syzygium rehderianum* | 0.00 | 0.00 | 0.00 | 0.00 | 0.40 | 3.67 |
| *Castanopsis hystrix* | 0.00 | 0.00 | 0.00 | 148.71 | 189.37 | 418.77 |
| *Michelia macclurel* | 0.00 | 0.00 | 0.00 | 19.83 | 0.00 | 0.00 |
| *Litsea glutinosa* | 0.00 | 0.00 | 0.00 | 9.12 | 10.76 | 1.38 |
| *Psychotria rubra* | 0.00 | 0.00 | 1.40 | 0.00 | 0.00 | 12.22 |
| *Tetradium glabrifolium* | 0.00 | 0.00 | 0.00 | 31.73 | 0.00 | 20.96 |
| *Pinus massoniana* | 0.08 | 0.00 | 0.00 | 0.00 | 0.00 | 0.00 |
| *Alangium kurzii* | 0.00 | 0.00 | 0.00 | 0.00 | 0.00 | 55.12 |
| *Glochidion eriocarpum* | 0.00 | 0.00 | 0.00 | 0.00 | 0.00 | 0.05 |
| *Ilex asprella* | 0.00 | 0.00 | 1.52 | 0.00 | 0.00 | 46.70 |
| *Eurya chinensis* | 0.00 | 0.00 | 0.00 | 0.00 | 0.00 | 1.33 |
| *Syzygium jambos* | 0.00 | 0.00 | 0.00 | 11.90 | 0.00 | 0.00 |
| *Toxicodendron vernicifluum* | 0.00 | 0.00 | 0.00 | 7.93 | 0.00 | 0.00 |
| *Evodia lepta* | 0.17 | 1.01 | 60.25 | 132.54 | 9.57 | 198.50 |
| *Litsea cubeba* | 0.00 | 0.00 | 11.79 | 65.63 | 4.62 | 447.18 |
| *Trema orientalis* | 0.03 | 4.82 | 0.00 | 0.00 | 0.00 | 0.00 |
| *Rhodomyrtus tomentosa* | 0.01 | 0.09 | 0.00 | 0.00 | 0.00 | 0.17 |
| *Schima Wallichii* | 4085.61 | 5813.81 | 10977.04 | 10978.06 | 12355.77 | 12055.64 |
| *Schefflera octophylla* | 0.00 | 0.00 | 0.00 | 0.00 | 0.00 | 0.46 |
| *Toxicodendron succedaneum* | 0.00 | 0.00 | 0.00 | 0.00 | 5.84 | 23.87 |
| *Cinnamomum burmanni* | 0.00 | 0.00 | 0.00 | 0.00 | 0.00 | 1.33 |
| *Aporosa dioica* | 0.00 | 0.00 | 0.00 | 0.00 | 0.59 | 3.74 |
| *Cinnamomum camphora* | 0.00 | 0.04 | 0.00 | 0.00 | 0.88 | 0.00 |
| *Podocarpus nagi* | 0.00 | 0.00 | 0.00 | 65.04 | 7.37 | 11.89 |
| *Carallia brachiata* | 0.00 | 0.00 | 0.00 | 0.00 | 0.00 | 0.52 |
| **Subtotal** | 4086.00 | 5819.88 | 11052.30 | 11900.04 | 13092.11 | 14192.15 |
|  |  |  |  |  |  |  |
| **Shrubs** |  |  |  |  |  |  |
| *Alangium chinense* | 0.00 | 0.00 | 0.00 | 0.93 | 0.00 | 0.00 |
| *Mallotus apelta* | 0.56 | 0.00 | 0.12 | 0.17 | 1.20 | 3.19 |
| *Mallotus paniculatus* | 0.00 | 0.38 | 0.00 | 0.00 | 1.26 | 0.00 |
| *Ficus variolosa* | 0.00 | 0.00 | 0.00 | 0.42 | 0.55 | 1.15 |
| *Litsea rotundifolia* | 5.18 | 11.05 | 1.75 | 6.82 | 7.79 | 5.90 |
| *Rhaphiolepis indica* | 3.46 | 5.69 | 7.13 | 21.20 | 7.17 | 30.48 |
| *Ficus hirta* | 0.00 | 0.00 | 0.00 | 5.03 | 0.06 | 3.15 |
| *Lagerstroemia* | 0.00 | 0.00 | 0.00 | 0.00 | 0.00 | 4.72 |
| *Baeckea frutescens* | 8.13 | 0.31 | 0.00 | 0.00 | 0.00 | 0.00 |
| *Clerodendron fortunatum* | 1.35 | 12.08 | 207.36 | 143.97 | 55.22 | 58.97 |
| *Schima superba* | 0.00 | 0.00 | 1.05 | 0.51 | 0.00 | 2.05 |
| *Breynia fruticosa* | 1.81 | 1.70 | 1.72 | 4.11 | 1.50 | 1.41 |
| *Syzygium rehderianum* | 0.00 | 0.00 | 0.00 | 0.00 | 0.00 | 3.98 |
| *Cyclosorus parasiticus* | 0.00 | 0.00 | 0.00 | 7.18 | 0.00 | 0.00 |
| *Gardenia jasminoides* | 7.03 | 1.67 | 4.67 | 16.61 | 7.26 | 19.14 |
| *Desmos chinensis* | 0.00 | 0.00 | 0.00 | 0.00 | 0.15 | 4.47 |
| *Litsea glutinosa* | 1.07 | 0.45 | 0.22 | 3.57 | 1.20 | 25.93 |
| *Psychotria rubra* | 5.44 | 8.91 | 29.45 | 59.76 | 41.73 | 233.59 |
| *Cymbidium* | 0.00 | 0.00 | 0.02 | 0.00 | 0.00 | 0.00 |
| *Radix Wikstroemae* | 8.01 | 2.85 | 0.68 | 1.64 | 0.00 | 0.00 |
| *Alangium kurzii* | 0.00 | 0.00 | 2.06 | 0.00 | 0.00 | 0.00 |
| *Glochidion eriocarpum* | 0.29 | 0.00 | 0.00 | 0.00 | 0.46 | 3.04 |
| *Ilex asprella* | 11.25 | 9.26 | 61.30 | 163.10 | 94.13 | 150.40 |
| *Eurya chinensis* | 1.32 | 4.23 | 12.72 | 13.07 | 27.47 | 28.87 |
| *Syzygium jambos* | 0.00 | 0.00 | 0.00 | 1.22 | 0.00 | 0.00 |
| *Evodia lepta* | 25.69 | 13.26 | 56.68 | 40.57 | 24.69 | 160.01 |
| *Litsea cubeba* | 0.00 | 1.40 | 9.36 | 15.49 | 5.48 | 5.66 |
| *Trema orientalis* | 0.00 | 0.00 | 0.17 | 0.00 | 4.19 | 2.05 |
| *Dianella ensifolia* | 0.00 | 0.00 | 0.10 | 0.00 | 0.00 | 0.00 |
| *Sapium discolor* | 0.00 | 0.00 | 0.00 | 0.00 | 0.28 | 0.00 |
| *Helicteres angustifolia* | 3.93 | 3.35 | 0.00 | 2.11 | 0.27 | 0.00 |
| *Glochidion puberum* | 2.16 | 0.35 | 0.63 | 0.26 | 0.00 | 0.00 |
| *Rhodomyrtus tomentosa* | 306.05 | 107.90 | 47.77 | 20.36 | 1.50 | 2.12 |
| *Blechnum orientale* | 0.00 | 0.00 | 151.31 | 83.84 | 0.00 | 0.00 |
| *Lindera communis* | 0.00 | 0.00 | 1.36 | 0.00 | 0.00 | 26.76 |
| *Urena lobata* | 0.00 | 0.00 | 2.16 | 1.72 | 4.30 | 1.77 |
| *Melastoma candidum* | 14.35 | 11.88 | 2.45 | 3.56 | 1.53 | 7.21 |
| *Schefflera octophylla* | 0.00 | 0.00 | 0.15 | 0.00 | 0.07 | 4.06 |
| *Acer fabri* | 0.00 | 0.00 | 0.00 | 1.30 | 0.00 | 0.00 |
| *Toxicodendron succedaneum* | 0.00 | 0.44 | 0.00 | 0.00 | 0.00 | 0.00 |
| *Cinnamomum burmanni* | 0.00 | 0.00 | 0.00 | 2.71 | 1.39 | 15.57 |
| *Aporosa dioica* | 0.00 | 0.00 | 0.00 | 0.00 | 0.00 | 4.38 |
| *Carallia brachiata* | 0.00 | 0.00 | 0.00 | 0.21 | 0.97 | 0.50 |
| **Subtotal** | 407.08 | 197.15 | 602.40 | 621.46 | 291.82 | 810.54 |
|  |  |  |  |  |  |  |
| **Herbs** |  |  |  |  |  |  |
| *smilax china* | 0.00 | 0.00 | 1.18 | 0.00 | 0.00 | 0.00 |
| *Mallotus apelta* | 0.15 | 0.00 | 0.00 | 0.00 | 0.00 | 0.00 |
| *Morinda parvifolia* | 0.01 | 0.00 | 0.00 | 0.00 | 0.00 | 0.00 |
| *Pteris semipinnata* | 0.00 | 0.00 | 0.00 | 0.00 | 0.00 | 1.32 |
| *Centella asiatica* | 0.02 | 0.00 | 0.00 | 0.00 | 0.00 | 0.00 |
| *Rhaphiolepis indica* | 0.67 | 0.38 | 1.35 | 0.00 | 0.00 | 0.00 |
| *Rubus alceaefolius* | 0.00 | 0.00 | 3.53 | 0.00 | 0.00 | 0.00 |
| *Torenia concolor* | 0.00 | 0.00 | 0.00 | 0.00 | 0.00 | 3.34 |
| *Lophatherum gracile* | 0.00 | 0.00 | 13.28 | 14.85 | 42.95 | 56.02 |
| *HerbaElephantopi* | 0.00 | 1.38 | 0.00 | 2.33 | 0.00 | 0.00 |
| *Melastoma dodecandrum* | 0.12 | 0.00 | 4.77 | 0.34 | 0.00 | 0.23 |
| *Eragrostis atrovirens* | 0.00 | 0.00 | 0.00 | 10.95 | 0.00 | 0.00 |
| *Panicum brevifolium* | 0.00 | 0.00 | 0.00 | 0.00 | 0.00 | 0.93 |
| *Pteris multifida* | 0.10 | 0.00 | 0.24 | 0.00 | 0.00 | 0.00 |
| *Cyrtococcum patens* | 26.88 | 0.15 | 0.00 | 0.00 | 0.00 | 0.00 |
| *Setaira viridis* | 3.15 | 2.02 | 0.18 | 0.00 | 0.00 | 0.00 |
| *Clerodendron fortunatum* | 20.38 | 1.10 | 18.23 | 21.42 | 0.00 | 0.00 |
| *Nvicrolepia hancei* | 0.00 | 0.00 | 0.00 | 0.00 | 0.00 | 3.40 |
| *Cyclosorus parasiticus* | 0.00 | 0.00 | 0.00 | 0.00 | 0.15 | 0.15 |
| *Gardenia jasminoides* | 1.42 | 1.88 | 2.34 | 0.00 | 0.00 | 0.00 |
| *Polygonum chinense* | 0.00 | 0.00 | 0.00 | 3.36 | 0.00 | 0.00 |
| *Psychotria rubra* | 0.00 | 0.00 | 0.00 | 8.96 | 0.00 | 0.00 |
| *Radix Wikstroemae* | 0.10 | 0.00 | 0.00 | 0.00 | 0.00 | 0.00 |
| *Miscanthus sinensis* | 0.00 | 0.00 | 0.00 | 0.00 | 1.34 | 3.86 |
| *Dicranopteris dichotoma* | 0.00 | 0.00 | 63.82 | 20.26 | 71.71 | 6.89 |
| *Ilex asprella* | 0.26 | 0.00 | 0.00 | 0.00 | 0.00 | 0.00 |
| *Eurya chinensis* | 1.26 | 14.37 | 0.00 | 2.91 | 0.00 | 0.00 |
| *Evodia lepta* | 2.82 | 11.11 | 0.00 | 11.42 | 0.00 | 0.00 |
| *Litsea cubeba* | 0.00 | 0.00 | 10.92 | 0.00 | 0.00 | 0.00 |
| *Dianella ensifolia* | 0.00 | 0.00 | 1.00 | 78.39 | 0.00 | 0.84 |
| *Helicteres angustifolia* | 2.33 | 3.65 | 4.19 | 0.00 | 0.00 | 0.00 |
| *Adiantum flabellulatum* | 0.00 | 0.11 | 12.05 | 13.93 | 0.91 | 5.79 |
| *Embelia laeta* | 0.00 | 0.00 | 41.57 | 0.00 | 0.00 | 0.00 |
| *Glochidion puberum* | 0.73 | 0.00 | 0.00 | 0.00 | 0.00 | 0.00 |
| *Rhodomyrtus tomentosa* | 3.71 | 0.37 | 3.56 | 11.52 | 0.00 | 0.00 |
| *Blechnum orientale* | 0.00 | 57.33 | 486.81 | 44.09 | 20.05 | 258.65 |
| *Nephrolepiscordifolia* | 0.00 | 0.00 | 0.00 | 39.78 | 0.00 | 0.00 |
| *Ishaemum indicum* | 3.09 | 0.00 | 0.00 | 0.00 | 0.00 | 0.00 |
| *Ottochloa nodosa* | 0.00 | 0.00 | 7.01 | 2.18 | 21.90 | 7.70 |
| *Urena lobata* | 0.00 | 0.00 | 6.34 | 0.00 | 0.00 | 0.00 |
| *Melastoma candidum* | 0.00 | 0.00 | 0.63 | 0.00 | 0.00 | 0.00 |
| *Lindsaea heterophylla* | 0.00 | 0.00 | 2.68 | 31.91 | 0.90 | 6.00 |
| *Cinnamomum burmanni* | 0.00 | 0.00 | 0.12 | 0.00 | 0.00 | 0.00 |
| *Mussaenda pubescens.* | 0.53 | 0.90 | 13.88 | 0.00 | 0.00 | 0.00 |
| *Lindsaea orbiculata* | 1.79 | 1.71 | 0.00 | 0.00 | 0.00 | 0.00 |
| **Sum** | 69.51 | 96.46 | 699.69 | 318.58 | 159.92 | 355.13 |
|  |  |  |  |  |  |  |
| **Total** | 4562.59 | 6113.49 | 12354.38 | 12840.09 | 13543.85 | 15357.82 |

Appendix D. Biomass composition in the *acacia* plantation (AP) during the study period (g/m^2^)

|  | Age | | | | | |
| --- | --- | --- | --- | --- | --- | --- |
| Latin Name | 11 | 13 | 19 | 21 | 23 | 26 |
| **Trees** |  |  |  |  |  |  |
| *Mallotus apelta* | 0.27 | 0.00 | 0.00 | 0.00 | 0.00 | 0.00 |
| *Litsea rotundifolia* | 0.00 | 0.00 | 0.00 | 1.32 | 4.66 | 21.11 |
| *Litsea glutinosa* | 0.00 | 0.00 | 0.00 | 0.00 | 3.14 | 869.96 |
| *Rhaphiolepis indica* | 0.00 | 0.00 | 0.00 | 0.22 | 0.00 | 5.62 |
| *Ficus hirta* | 0.05 | 0.00 | 0.00 | 0.00 | 0.00 | 0.00 |
| *Syzygium rehderianum* | 0.00 | 0.00 | 0.00 | 0.00 | 5.45 | 0.00 |
| *Castanopsis hystrix* | 0.00 | 0.00 | 0.00 | 0.00 | 0.00 | 1.82 |
| *Dioscorea bulbifera* | 1.26 | 0.00 | 0.00 | 0.00 | 0.00 | 0.00 |
| *Gardenia jasminoides* | 0.12 | 0.00 | 0.00 | 0.45 | 0.00 | 0.00 |
| *Psychotria rubra* | 0.09 | 0.00 | 0.00 | 0.00 | 0.07 | 2370.41 |
| *Melia azedarach* | 0.00 | 0.00 | 0.00 | 0.00 | 0.08 | 0.00 |
| *Tetradium glabrifolium* | 239.53 | 181.65 | 357.24 | 0.00 | 18.70 | 90.19 |
| *Radix wikstroemae* | 0.00 | 0.00 | 0.00 | 0.66 | 0.00 | 2.38 |
| *Pinus massoniana* | 25.75 | 16.12 | 0.00 | 0.00 | 0.00 | 622.19 |
| *Acacia mangium* | 4570.20 | 5607.48 | 12681.94 | 14063.05 | 14679.39 | 10105.59 |
| *Ilex asprella* | 4.41 | 0.00 | 0.00 | 175.69 | 0.00 | 59.20 |
| *Eurya chinensis* | 0.00 | 0.00 | 0.00 | 0.00 | 0.00 | 6.75 |
| *Toxicodendron vernicifluum* | 0.00 | 2.36 | 12.27 | 0.00 | 0.00 | 0.00 |
| *Evodia lepta* | 30.94 | 0.76 | 60.88 | 0.00 | 0.33 | 0.65 |
| *Ilex triflora* | 18.28 | 0.00 | 0.00 | 0.00 | 0.00 | 0.00 |
| *Litsea cubeba* | 41.01 | 32.58 | 16.89 | 0.00 | 24.62 | 111.41 |
| *Trema orientalis* | 0.18 | 0.00 | 0.00 | 0.00 | 0.00 | 0.00 |
| *Rhodomyrtus tomentosa* | 0.00 | 0.00 | 0.00 | 1.98 | 0.00 | 12.60 |
| *Lindera communis* | 0.00 | 0.00 | 0.00 | 0.00 | 0.00 | 13.39 |
| *Toxicodendron succedaneum* | 7.42 | 0.00 | 0.00 | 0.00 | 0.33 | 1098.01 |
| *Cinnamomum burmanni* | 0.00 | 0.00 | 0.00 | 0.00 | 0.25 | 9.64 |
| **Subtotal** | 4939.50 | 5840.96 | 13129.23 | 14243.36 | 14737.01 | 15400.93 |
|  |  |  |  |  |  |  |
| **Shrubs** |  |  |  |  |  |  |
| *Mallotus apelta* | 0.00 | 0.00 | 0.00 | 0.23 | 0.00 | 0.00 |
| *Ficus variolosa* | 0.00 | 0.00 | 0.00 | 0.55 | 8.04 | 18.31 |
| *Camellia sinensis* | 0.00 | 0.00 | 0.00 | 1.17 | 0.00 | 0.00 |
| *Litsea rotundifolia* | 0.00 | 0.00 | 0.00 | 4.52 | 0.19 | 28.17 |
| *Litsea glutinosa* | 0.00 | 0.00 | 0.00 | 2.80 | 1.45 | 53.58 |
| *Rhaphiolepis indica* | 0.00 | 0.00 | 3.58 | 2.95 | 0.02 | 38.24 |
| *Ficus hirta* | 0.00 | 0.27 | 0.07 | 0.67 | 0.02 | 19.56 |
| *Pterospermum heterophyllum* | 0.00 | 0.00 | 0.22 | 0.00 | 0.00 | 0.00 |
| *Clerodendron fortunatum* | 1.07 | 2.70 | 18.59 | 1.95 | 4.18 | 10.75 |
| *Syzygium hainanense* | 0.00 | 0.00 | 0.00 | 0.00 | 0.00 | 6.25 |
| *Breynia fruticosa* | 0.00 | 0.00 | 0.09 | 0.68 | 0.05 | 19.12 |
| *Aralia decaisneana* | 0.00 | 0.00 | 0.00 | 0.26 | 0.00 | 0.00 |
| *Cratoxylon ligustrinum* | 0.40 | 0.13 | 0.00 | 0.00 | 0.00 | 0.00 |
| *Gardenia jasminoides* | 0.36 | 0.38 | 1.22 | 4.09 | 0.99 | 12.36 |
| *Desmos chinensis* | 0.00 | 0.00 | 0.00 | 0.00 | 0.00 | 19.09 |
| *Psychotria rubra* | 0.00 | 0.00 | 1.86 | 3.72 | 1.68 | 28.57 |
| *Tetradium glabrifolium* | 0.00 | 0.00 | 0.00 | 0.00 | 0.00 | 36.83 |
| *Radix Wikstroemae* | 0.00 | 0.00 | 0.00 | 7.69 | 0.00 | 29.61 |
| *Glochidion eriocarpum* | 0.00 | 0.00 | 0.00 | 0.00 | 0.00 | 4.02 |
| *Ilex asprella* | 2.76 | 1.06 | 54.53 | 64.50 | 112.55 | 163.62 |
| *Eurya chinensis* | 6.42 | 2.46 | 47.86 | 10.53 | 3.58 | 66.43 |
| *Aglaia odorata* | 0.00 | 0.00 | 0.00 | 0.00 | 0.00 | 15.40 |
| *Toxicodendron vernicifluum* | 0.00 | 0.00 | 7.38 | 3.99 | 0.00 | 0.00 |
| *Evodia lepta* | 0.05 | 0.03 | 8.44 | 7.32 | 5.88 | 11.39 |
| *Ilex triflora* | 0.00 | 0.00 | 63.32 | 0.00 | 0.00 | 0.00 |
| *Litsea cubeba* | 0.00 | 0.00 | 31.48 | 5.69 | 1.95 | 35.70 |
| *Trema orientalis* | 0.00 | 0.00 | 1.69 | 0.00 | 0.00 | 0.00 |
| *Sapium discolor* | 0.00 | 0.00 | 0.48 | 0.00 | 0.00 | 0.00 |
| *Helicteres angustifolia* | 0.00 | 0.00 | 0.00 | 0.51 | 0.00 | 1.45 |
| *Geum aleppicum* | 0.00 | 0.00 | 0.02 | 0.00 | 0.00 | 0.00 |
| *Glochidion puberum* | 0.14 | 0.03 | 0.00 | 0.00 | 0.00 | 0.00 |
| *Rhodomyrtus tomentosa* | 0.40 | 0.18 | 0.13 | 16.53 | 7.52 | 34.41 |
| *Blechnum orientale* | 0.00 | 0.00 | 0.45 | 11.05 | 0.00 | 0.00 |
| *Lindera communis* | 0.00 | 0.00 | 0.00 | 0.00 | 0.00 | 37.12 |
| *Urena lobata* | 5.00 | 0.00 | 0.00 | 0.00 | 0.00 | 0.00 |
| *Melastoma candidum* | 0.08 | 0.00 | 1.72 | 1.23 | 0.00 | 12.50 |
| *Toxicodendron succedaneum* | 0.00 | 0.00 | 0.52 | 0.00 | 0.00 | 70.32 |
| *Cinnamomum burmanni* | 0.00 | 0.00 | 0.00 | 1.04 | 0.12 | 27.57 |
| *Cinnamomum camphora* | 0.00 | 0.00 | 0.00 | 0.00 | 0.00 | 16.07 |
| **Subtotal** | 16.70 | 7.23 | 243.65 | 153.64 | 148.21 | 816.44 |
|  |  |  |  |  |  |  |
| **Herbs** |  |  |  |  |  |  |
| *Rubus alceaefolius* | 0.00 | 0.00 | 5.97 | 0.00 | 0.00 | 0.00 |
| *Lophatherum gracile* | 0.00 | 0.00 | 0.00 | 9.57 | 0.00 | 18.04 |
| *Melastoma dodecandrum* | 0.00 | 0.00 | 0.00 | 0.00 | 0.38 | 0.00 |
| *Pteris multifida* | 0.21 | 0.00 | 0.00 | 0.00 | 0.00 | 0.00 |
| *Cyrtococcum patens* | 13.53 | 35.07 | 0.00 | 0.00 | 0.00 | 0.00 |
| *Clerodendron fortunatum* | 0.00 | 22.67 | 24.17 | 5.51 | 0.00 | 0.00 |
| *Cyclosorus parasiticus* | 0.00 | 0.00 | 0.00 | 1.16 | 0.05 | 0.00 |
| *Gardenia jasminoides* | 0.00 | 0.00 | 12.17 | 0.00 | 0.00 | 0.00 |
| *Polygonum chinense* | 0.00 | 0.00 | 12.39 | 0.00 | 0.00 | 0.00 |
| *Pteris ensiformis* | 0.00 | 0.00 | 0.00 | 0.00 | 0.00 | 6.76 |
| *Psychotria rubra* | 0.00 | 27.06 | 0.00 | 0.00 | 0.00 | 0.00 |
| *Spermacoce latifolia* | 0.00 | 0.00 | 7.61 | 0.00 | 0.00 | 0.00 |
| *Radix Wikstroemae* | 0.00 | 0.00 | 9.58 | 11.98 | 0.00 | 0.00 |
| *Miscanthus sinensis* | 0.00 | 0.00 | 0.00 | 388.77 | 5.64 | 338.20 |
| *Dicranopteris dichotoma* | 6.14 | 54.11 | 8.26 | 80.88 | 854.57 | 402.09 |
| *Evodia lepta* | 0.00 | 0.00 | 0.00 | 13.66 | 0.00 | 0.00 |
| *Litsea cubeba* | 0.00 | 5.07 | 0.00 | 0.00 | 0.00 | 0.00 |
| *Dianella ensifolia* | 0.00 | 0.00 | 0.00 | 39.81 | 0.00 | 5.64 |
| *Hyptis suaveolens* | 36.08 | 0.00 | 0.00 | 0.00 | 0.00 | 0.00 |
| *Adiantum flabellulatum* | 13.08 | 0.00 | 0.00 | 3.01 | 7.75 | 6.76 |
| *Paspalum distichum* | 0.00 | 0.00 | 0.00 | 0.00 | 0.00 | 57.49 |
| *Embelia laeta* | 0.00 | 0.00 | 0.88 | 0.00 | 0.00 | 0.00 |
| *Glochidion puberum* | 64.94 | 0.00 | 1.41 | 0.00 | 0.00 | 0.00 |
| *Blechnum orientale* | 0.00 | 0.00 | 136.08 | 342.17 | 167.60 | 362.13 |
| *Ishaemum indicum* | 4.33 | 0.00 | 0.00 | 0.52 | 4.04 | 0.00 |
| *Ottochloa nodosa* | 0.00 | 0.00 | 110.72 | 7.85 | 16.68 | 59.19 |
| *Urena lobata* | 0.00 | 0.00 | 1.68 | 0.00 | 0.00 | 0.00 |
| *Mussaenda pubescens* | 0.18 | 0.00 | 14.70 | 0.00 | 0.00 | 0.00 |
| **Subtotal** | 138.48 | 143.98 | 345.62 | 904.89 | 1056.70 | 1256.31 |
|  |  |  |  |  |  |  |
| **Total** | 5094.67 | 5992.18 | 13718.50 | 15301.89 | 15941.93 | 17473.68 |

Appendix E. Community characteristics of the three plantations.

| **Item** | **Age** | | | | | |
| --- | --- | --- | --- | --- | --- | --- |
|  | **11** | **13** | **19** | **21** | **23** | **26** |
| **Mixed native plantation (NP)** | |  |  |  |  |  |
| Investigation Area (m^2^) | 400 | 400 | 400 | 900 | 900 | 900 |
| Tree MH (m) | 4.8 | 3.4 | 7.3 | 7.0 | 8.4 | 4.3 |
| Tree MDBH (cm) | 2.7 | 4.1 | 7.2 | 9.5 | 9.8 | 5.2 |
| *Schima Wallichii* MH (m) | 5.3 | 5.9 | 9.6 | 9.4 | 11.7 | 9.3 |
| *Schima Wallichii* MDBH (cm) | 9.2 | 10.7 | 13.4 | 14.1 | 14.6 | 15.9 |
| Family | 19 | 20 | 26 | 32 | 28 | 32 |
| Genus | 28 | 27 | 39 | 42 | 40 | 45 |
| Specie | 30 | 28 | 41 | 46 | 44 | 48 |
| Ferns | 1 | 2 | 5 | 6 | 5 | 6 |
| Gymnosperm | 1 | 0 | 0 | 1 | 1 | 1 |
| Angiosperm | 28 | 26 | 36 | 39 | 38 | 41 |
| **Conifer plantation (CP)** | |  |  |  |  |  |
| Investigation Area (m^2^) | 400 | 400 | 400 | 900 | 900 | 900 |
| Tree MH (m) | 2.79 | 4.2 | 8.09 | 9.64 | 9.08 | 5.41 |
| Tree MDBH (cm) | 3.01 | 4.95 | 9.7 | 8.43 | 9.55 | 6.57 |
| *Pinus massoniana* M H (m) | 4.01 | 5.5 | 9.12 | 13.98 | 11.99 | 10.23 |
| *Pinus massoniana* M DBH (cm) | 7.15 | 8.42 | 11.93 | 10.69 | 13.98 | 14.83 |
| *Cunninghamia lanceolata* |  |  |  |  |  |  |
| MH (m) | 3.13 | 4.03 | 7.58 | 7.77 | 9.24 | 8.55 |
| MDBH (cm) | 3.61 | 4.84 | 8.59 | 8.24 | 9.25 | 11.38 |
| Family | 21 | 16 | 27 | 25 | 27 | 22 |
| Genus | 29 | 20 | 35 | 32 | 34 | 37 |
| Species | 31 | 24 | 38 | 36 | 35 | 38 |
| Ferns | 3 | 2 | 6 | 5 | 5 | 6 |
| Gymnosperm | 3 | 3 | 2 | 2 | 2 | 2 |
| Angiosperm | 25 | 19 | 30 | 29 | 28 | 30 |
| ***Acacia mangium* plantation (AP)** | |  |  |  |  |  |
| Investigation Area (m^2^) | 400 | 400 | 400 | 3800 | 2400 | 1600 |
| Tree MH (m) | 6.89 | 7.02 | 9.99 | 11.19 | 9.35 | 5.37 |
| Tree MDBH (cm) | 10.08 | 10.87 | 13.96 | 17.3 | 12.25 | 6.58 |
| *Acacia mangium* MH (m) | 8.6 | 9.2 | 12.3 | 13.2 | 13 | 12.5 |
| *Acacia mangium* MDBH (cm) | 13.7 | 15 | 15.6 | 18.9 | 17.7 | 18.3 |
| Family | 21 | 14 | 20 | 23 | 21 | 26 |
| Genus | 25 | 15 | 28 | 31 | 27 | 34 |
| Species | 28 | 17 | 31 | 33 | 31 | 38 |
| Ferns | 3 | 1 | 1 | 4 | 4 | 5 |
| Gymnosperm | 1 | 1 | 0 | 0 | 0 | 1 |
| Angiosperm | 24 | 15 | 30 | 29 | 27 | 32 |

MH - Mean height; MDBH - Mean diameter at breast height.
